# Identification of *Jun* loss promotes resistance to histone deacetylase inhibitor entinostat through Myc signaling in luminal breast cancer

**DOI:** 10.1186/s13073-018-0597-3

**Published:** 2018-11-30

**Authors:** Maki Tanioka, Kevin R. Mott, Daniel P. Hollern, Cheng Fan, David B. Darr, Charles M. Perou

**Affiliations:** 1 Lineberger Comprehensive Cancer Center, University of North Carolina at Chapel Hill, Chapel Hill, NC 27599 USA; 2 Department of Genetics, University of North Carolina, Chapel Hill, NC 27599 USA; 3 Lineberger Comprehensive Cancer Center, The Animal Study Core, University of North Carolina at Chapel Hill, Chapel Hill, NC 27599 USA

## Abstract

**Background:**

Based on promising phase II data, the histone deacetylase inhibitor entinostat is in phase III trials for patients with metastatic estrogen receptor-positive breast cancer. Predictors of sensitivity and resistance, however, remain unknown.

**Methods:**

A total of eight cell lines and nine mouse models of breast cancer were treated with entinostat. Luminal cell lines were treated with or without entinostat at their IC_50_ doses, and MMTV/Neu luminal mouse tumors were untreated or treated with entinostat until progression. We investigated these models using their gene expression profiling by microarray and copy number by arrayCGH. We also utilized the network-based DawnRank algorithm that integrates DNA and RNA data to identify driver genes of resistance. The impact of candidate drivers was investigated in The Cancer Genome Atlas and METABRIC breast cancer datasets.

**Results:**

Luminal models displayed enhanced sensitivity to entinostat as compared to basal-like or claudin-low models. Both in vitro and in vivo luminal models showed significant downregulation of *Myc* gene signatures following entinostat treatment. *Myc* gene signatures became upregulated on tumor progression in vivo and overexpression of *Myc* conferred resistance to entinostat in vitro. Further examination of resistance mechanisms in MMTV/Neu tumors identified a portion of mouse chromosome 4 that had DNA copy number loss and low gene expression. Within this region, *Jun* was computationally identified to be a driver gene of resistance. *Jun* knockdown in cell lines resulted in upregulation of *Myc* signatures and made these lines more resistant to entinostat. *Jun*-deleted samples, found in 17–23% of luminal patients, had significantly higher *Myc* signature scores that predicted worse survival.

**Conclusions:**

Entinostat inhibited luminal breast cancer through Myc signaling, which was upregulated by *Jun* DNA loss to promote resistance to entinostat in our models. *Jun* DNA copy number loss, and/or high MYC signatures, might represent biomarkers for entinostat responsiveness in luminal breast cancer.

**Electronic supplementary material:**

The online version of this article (10.1186/s13073-018-0597-3) contains supplementary material, which is available to authorized users.

## Background

Despite advances in early detection and perioperative treatments, breast cancer remains the second leading cause of cancer deaths among women in developed countries [1]. This is because a certain portion of the patients continue to develop fatal metastatic disease and eventually succumb to death. Estrogen receptor (ER)-positive tumors comprise 70% of the breast cancer population and most of them respond to aromatase inhibitors (AIs); however, others acquire resistance. Currently, the combinations of CDK4/6 inhibitors, or a mTOR inhibitor with AIs, have shown improvement of progression-free survival in patients with metastatic ER-positive breast cancer [2–4]. Unlike these agents, entinostat, a class I histone deacetylase (HDAC) inhibitor, may augment response to endocrine therapy. HDAC inhibitors have been thought to induce histone acetylation leading to transcriptional re-activation of epigenetically inactivated cancer-associated genes which suppress cell proliferation and promote apoptosis [5]. Class I HDAC isozymes include HDAC1–3, whose expression has been shown to be increased in hormone receptor-positive or high-grade tumors [6]. Indicating the importance of epigenetic modification in the context of endocrine therapy, entinostat with letrozole combination therapy was able to reduce the volume of letrozole-resistant tumors [7]. From a safety standpoint, entinostat does not target class II HDACs which are expressed in the heart [8]; therefore, this selectivity of entinostat may eliminate serious side effects such as QT-prolongation and cardiac infarction that have been associated with pan-HDAC inhibitors [9, 10]. For postmenopausal women with metastatic ER-positive breast cancer, a randomized phase II trial of entinostat showed benefits in both progression-free and overall survival and the incidence of reported cardiac disorders was similar between entinostat and placebo arms [11]. With these promising results, entinostat received breakthrough designation from the Food and Drug Administration (FDA), and currently, a phase III registration trial E2112 (NCT02115282) is ongoing.

Some HDAC inhibitors, Vorinostat, Panobinostat, Belinostat, and Romidepsin, have been granted FDA approval for cancer, yet there are no validated markers for their clinical decision making. Although c-Myc has been shown to be a key target for sensitivity to other HDAC inhibitors in various cancers [12–15], it is still unclear whether absolute transcriptional or genomic levels of c-Myc (hereafter, Myc) predict sensitivity to all HDAC inhibitors. In addition, Myc remains difficult to target with small molecule Myc inhibitors despite numerous attempts. As for entinostat in breast cancer, a number of molecular features have been suggested as underlying mechanisms of response. This includes upregulation of ER [7], downregulation of Akt [16], upregulation of E-cadherin [17], and functional inhibition of myeloid-derived suppressor cells [18, 19]. However, studies detailing tumor responses to entinostat are still needed to identify modes of sensitivity and resistance. Here we studied entinostat’s mechanism of action using multiple models of luminal and non-luminal breast cancers and identified two possible biomarkers of resistance.

## Methods

### Breast cancer cell lines

The human breast cancer cell lines were maintained in standard growth media (SKBR3, BT474, MCF-7, T47D) in RPMI (Gibco) plus 10% FBS (Sigma) and anti-biotic anti-mycotic (Gibco) or in DMEM (Gibco, high glucose) with 10% FBS (MDA231) [20]. Hs578t and WHIM12 cell lines were cultured in HuMEC media with supplements (Gibco) plus Bovine Pituitary Extract (Gibco) and anti-biotic anti-mycotic (Gibco). WHIM12 is a patient-derived xenograft cell line obtained from Matthew Ellis (Baylor College of Medicine). All cell lines were grown at 37 °C and 5% carbon dioxide and were obtained from the American Type Culture Collection unless otherwise specified.

### Cell proliferation assay

The effects of entinostat on the proliferation of various human or mouse cancer cell lines were determined by using the [3-(4,5-dimethylthiazol-2-yl)-5-(3-carboxymethoxyphenyl)-2-(4-sulfophenyl)2H-tetrazolium [21] assay (CellTiter 96® AQueous One Solution Cell Proliferation Assay, Promega), except for T47D where the effects were measured using Cell titer glo (Promega). A total of 3000–5000 cells were seeded into 96-well culture plates and treated with entinostat (Sigma) for 72 h and then treated with MTS for 2 h. Cell viability was determined by measuring the absorbance at 490 nm. Six replicates for each time point were measured. The generation of dose-response curves was performed using the GraphPad Prism.

### Genetically engineered mouse models (GEMMs)

GEMMs of strain *FVB* carrying a transgene for rat HER2 (MMTV/Neu) [22] and C3 SV40 T-antigen (C3tag) [23], or patient-derived xenografts (PDXs) WHIM8 and WHIM35 [24] in Nod-Skid-Gamma (NSG) mice were bred in-house and observed until the onset of a mammary tumor approximately 0.6 cm in any dimension. Tumors derived from TP53 −/− mammary gland transplant lines (T2, T11, 2225L, 2208L, and 2396R) [25] were passaged in BALB/c wild-type mice by subcutaneous injection of one half million cells resuspended in Matrigel into the mammary fat pad as previously described [26]. A minimum tumor volume of approximately 0.6 cm in size was used as the starting point for treatment studies; 12–28 mice for each experiment were randomized into untreated or treated groups and monitored with tumor growth measurements (Additional file 1: Figure S1). For C3tag and MMTV/Neu mice, we added information on 30 or more mice using historical controls, as well as contemporaneous controls. Tumor volumes were measured by caliper as (width) × (length)^2^/2 for MMTV/Neu or C3tag and π/6 × (length)^3^ for transplant lines. Entinostat (Sigma) was milled into chow at 12 mg/kg, which was determined by in-house dose-finding study of entinostat. For short-term response, tumor size was measured at baseline for all the models and at 7-day point for 2225L, 10-day point for 2396R and T2, 14-day point for 2208L, and 21-day point for WHIM8, WHIM35, T11, C3tag, and MMTV/Neu. Treatment periods were dependent on inherent tumor line growth rates, and the percent change in volume was used to quantify response. For survival, treatment with entinostat was started at time zero (i.e., tumor 0.6 cm) and continued until either the mouse developed a tumor burden sufficient to warrant euthanasia (2 cm in any dimension) or until weight loss totaling 20% of the initial starting body mass was observed. All work was done under protocols approved by the University of North Carolina (UNC, Chapel Hill, NC) Institutional Animal Care and Use Committee.

### DNA, RNA, and protein extraction

Total RNA was purified using the RNeasy Mini kit (Qiagen), and DNA was extracted using the DNeasy Kit (Qiagen). We extracted nuclear protein using NE-PER nuclear and cytoplasmic extraction reagents (Thermo Scientific). Following extraction, protein concentration was determined using Micro BCA protein assay kit (Thermo Scientific).

### Gene expression and signature analysis

A total of 1 μg human RNA or 2 μg of mouse RNA were purified and profiled as described previously using DNA oligo microarrays (Agilent Technologies, USA) [27]. The LOWESS normalized log2 ratios (Cy5 sample/Cy3 control) of probes mapping to the same gene (Entrez ID) were averaged to generate gene-level expression estimates. Gene expression changes were measured in luminal cell lines SKBR3 (ER-/HER2+), BT474 (ER+/HER2+), and MCF7 (ER+/HER2-) treated with or without entinostat at their IC_50_ doses. Significantly differentially expressed genes were identified (false discovery rate of 0%) using unpaired two-class significance analysis of microarrays (SAM) [28] to compare untreated vs. entinostat-treated samples. Hierarchical clustering using 814 of The Genome Cancer Atlas [29] breast cancer samples was done using Gene Cluster 3.0 [30]. For hierarchical clustering analyses, the genes/rows were median centered, and clustering of arrays was conducted with correlation centered genes and arrays, and centroid linkage. Array cluster viewing and display were conducted using JavaTreeview v1.1. 5r2 [31]. We defined “signatures” as any gene set that contained a minimum of 10 genes and a Pearson node correlation greater than 0.5 in this TCGA breast cancer hierarchical cluster dataset.

We investigated the significance of gene signatures using the “Investigate Gene Sets” method of Gene Set Enrichment Analysis (GSEA, http://software.broadinstitute.org/gsea/msigdb/annotate.jsp) [32]. We also applied a collection of 517 publicly available, gene expression signatures, representing multiple biological pathways and cell types as well as entinostat signatures [33]. These 517 signatures have all been published [34] and obtained from 73 publications or GSEA [32] and partially summarized by Fan et al. [35]. Using the TCGA data, we applied each signature to the dataset in a manner consistent with their derivation. For 480 signatures with homogeneous expression across genes, we used the median expression value from all genes within a signature, and 37 signatures were based on correlation to predetermined gene centroids or based on pre-specified published algorithms.

Using the gene expression data from SKBR3, BT474, and MCF-7 treated with or without entinostat, we also investigated three *Myc* gene signatures which represents Myc signaling: MYC.1PFDR_UP [21] and MYC.2012 [36] derived from breast cancer mouse models comparing MMTV-Myc and other models, and DUKE.MODULE13 [37] comparing Myc-overexpressed versus control human mammary epithelial cells. Genes in mouse and human version of signatures are shown in Additional file 2: Table S1. We used the median expression value from all genes in each signature as a gene signature score.

In addition, a total of 27 MMTV-Neu mouse tumors (luminal subtype) were untreated (*N* = 8) or treated with entinostat at 12 mg/kg for 3 weeks (*N* = 5), 6 weeks (*N* = 6), or until progression after complete response (*N* = 8). We profiled gene expression of these tumors and calculated *MYC* gene signature scores for each subgroup. Using the Mouse Genome Database (http://www.informatics.jax.org/homology.shtml), the lists of human *MYC* gene signatures were converted to orthologous mouse genes.

### Lentiviral transfection

To determine whether Myc or Jun levels influence the effects of entinostat on cell viability, we transfected luminal breast cancer cells with lentiviruses expressing the non-degradable, phosphorylation mutant (T58A) of *Myc* or two kinds of shRNA targeting *Jun*. To overexpress MycT58A or mCherry, constructs in pLEX-MCS-puro vector (Thermo Scientific) were kindly provided by Gary L Johnson [9]. HEK293T cells were transfected with Lipofectamine 2000 (Invitrogen) with pLEX-MCS-puro constructs and packaging plasmids. Forty-eight hours after transfection, viral supernatant was collected and filtered through 0.45-μm syringe filters. To knockdown *Jun* expression, shRNA for *Jun* and scramble DNA as a control were obtained from Cyagen and the pLV-Puro-U6 vector was used. Target sequences for *Jun* knockdown were ATTCGATCTCATTCAGTATTA or TTCTGGCCTGCCTTCGTTAAC at 3′UTR of *Jun* (i.e., two different RNAi targeting constructs). After SKBR-3 or MCF-7 cells were transduced with lentivirus-mediated mutant *Myc* with in the presence of 6 μg/ml polybrene, the cells were incubated in various doses of entinostat for 72 h, and then the viability of cells was measured using the MTS assay. Likewise, BT474 or T47D cells were transfected with lentivirus-mediated shRNA for *Jun*.

Expression levels of Myc or Jun protein in nucleus were determined by Western blot following recommendations of antibody suppliers. Antibodies used were HA-tag for exogenous Myc (C29F4, Cell Signaling), endogenous Myc (D3N8F, Cell Signaling), Jun (60A8, Cell Signaling), and beta-actin (#4967, Cell Signaling) as a housekeeping gene. The relative chemiluminescent intensities were quantified in individual frames using ImageJ software (NIH).

### Assessment of DNA copy number changes in MMTV/Neu tumors

To investigate DNA copy number changes, we used the custom HD-array Comparative Genomic Hybridization (aCGH) platform which was designed and built on the Mouse 244 k Custom Oligo platform (GPL15359 Agilent UNC Perou Lab 1 × 244 k Custom Tiling CGH Array). Two hundred thirty thousand six hundred and six probes cover a total region of 45 Mb, and this design gives an average resolution of 200 bp between contiguous probes. Labeling and hybridization were performed according to the manufacturer’s instructions using the Agilent Genomic DNA Labeling Kit PLUS (Catalog Number 5188–5309). One microgram of DNA from liver or spleen of FVB strain mouse was used as normal reference DNA, which was compared versus 1 μg of DNA from every mouse tumor sample. Microarrays were scanned on an Agilent DNA Microarray scanner (G2565CA) and the data uploaded to the University of North Carolina Microarray Database (www.genome.unc.edu). To determine regions of Copy Number Aberration (CNA), we utilized the R package SWITCHdna [38], which can identify breakpoints in aCGH data. SWITCHdna detects transition points that maximize the *F* statistic and have regions on either side of the breakpoint that are larger than 250 kb. Following detection of the transition points, a log2-ratio segment average value and corresponding *z*-score are determined, along with the number of observations used. In this study, we used a z-score of 3 and a minimum intensity measurement of 0.09. The end results are the identification of segments of CNA, along with a quantitative value for that copy number change (i.e., loss or gain). All subsequent plots were produced after applying this significance filter to our data. These segment-level copy number values were changed into gene level using R package Switchplus [39].

### DawnRank analysis

We used the DawnRank algorithm [40] as a novel computational method that uses within-tumor integrated analyses of DNA aberrations in context of RNA expression that is used to population predetermined protein-protein networks in order to find possible individual driver genes that might predict resistance to entinostat. Using the DawnRank predefined protein-protein interaction networks, we populated this network with mRNA gene expression data for each sample and calculated a score for each gene based upon expression of the genes connected to it in the network. Using somatically altered genes with CNA data described above, we applied DawnRank to four groups of the MMTV-Neu mouse tumors which were untreated (*N* = 6), or treated with entinostat at 12 mg/kg for 3 weeks (*N* = 4), 6 weeks (*N* = 4), or until progression after complete response (*N* = 8) according to the “percentrank” analysis mode, which aggregates the DawnRank results across a predefined set of samples.

### TCGA and METABRIC samples

Two independent publicly available human breast cancer datasets, TCGA [29] and METABRIC [41], were investigated to validate the impact of *Jun* deletion. We used PAM50 intrinsic subtypes, mRNA expression, and DNA copy number changes in 814 primary breast tumors from TCGA. PAM50 intrinsic subtypes and mRNA expression data are publicly available at cBioPortal website (http://cancergenome.nih.gov/). Copy number variation across the genome was determined as follows: The sequence reads were aligned to the genome (hg 19) using the bwa mem algorithm (https://github.com/lh3/bwa; v0.7.4) with the default parameters. Duplicates were removed using Picard (http://broadinstitute.github.io/picard/). Quality statistics were also generated with Picard including measures of fragment length, sequence content, alignment, capture bias and efficiency, coverage, and variant call metrics. Copy number assessments were performed using SynthEx [42]. In brief, counts data for fixed 100-kb bin were generated using BEDTools [43]. The read ratios were calculated using the “synthetic normal” strategy described in SynthEx. A trending filter procedure was applied to segment the genome. The segment-level copy number values, which is the log2 ratios of normalized signal intensities between tumor and reference, were finally corrected by purity and ploidy estimates from SynthEx, taking whole genome doubling into account for these values. These segment-level values were changed into gene-level using Switchplus [39]. Copy number values derived from exome sequencing were compared with those from SNP6.0 among the TCGA samples [44] with ploidy 1.75–2.5, then the thresholds for gain or loss were determined as 0.25 or − 0.32, respectively [42]; we applied these thresholds to copy number values on the TCGA samples to call gained and lost segments. All genomic data, including DNA copy number and gene expression, have been deposited into the GEO (series ID GSE118744).

The METABRIC human breast cancer dataset includes breast cancer-specific survival data as well as gene expression and DNA copy number data of 1992 resected primary breast tumors. All clinical and genomic data are also publicly available at the cBioPortal. Copy number datasets within the portal are generated by GISTIC [45] to determine the copy number status of each gene in each sample. Amplification or deletion was determined by applying both low- (− 1, − 2) and high-level (+ 1, + 2) thresholds to the gene copy levels of all the samples. In our study, *Jun* copy number loss was defined by “− 2” (possibly a homozygous deletion) and “− 1” (possibly a heterozygous deletion). To investigate the clinical impact of three Myc signatures (Myc_1pFDR, Myc.2012, or Duke_Module13_myc), the patients with luminal A or luminal B PAM50 subtype [46] breast cancers were classified into three rank order groups according to scores from each Myc signature. To investigate clinical impact of *Jun* copy number loss, the patients were classified into groups with/without *Jun* copy number loss, and breast cancer-specific survival was assessed by Kaplan-Meier curves. Patients with survival of > 20 years were excluded. Two-sided log-rank tests and univariate Cox regression analyses were conducted to determine significance of each endpoint.

Statistical analyses on signature scores, box plots, SAM, DawnRank, and Kaplan-Meier curves of human samples were performed using R version 3.1.2, while generation of survival curves or response plots on mouse models, and IC_50_ curves on cell lines were performed using the GraphPad Prism version 7 software (GraphPad Software, San Diego).

## Results

### Sensitivity to entinostat among breast cancer models in vivo and in vitro

**Fig. 1:**
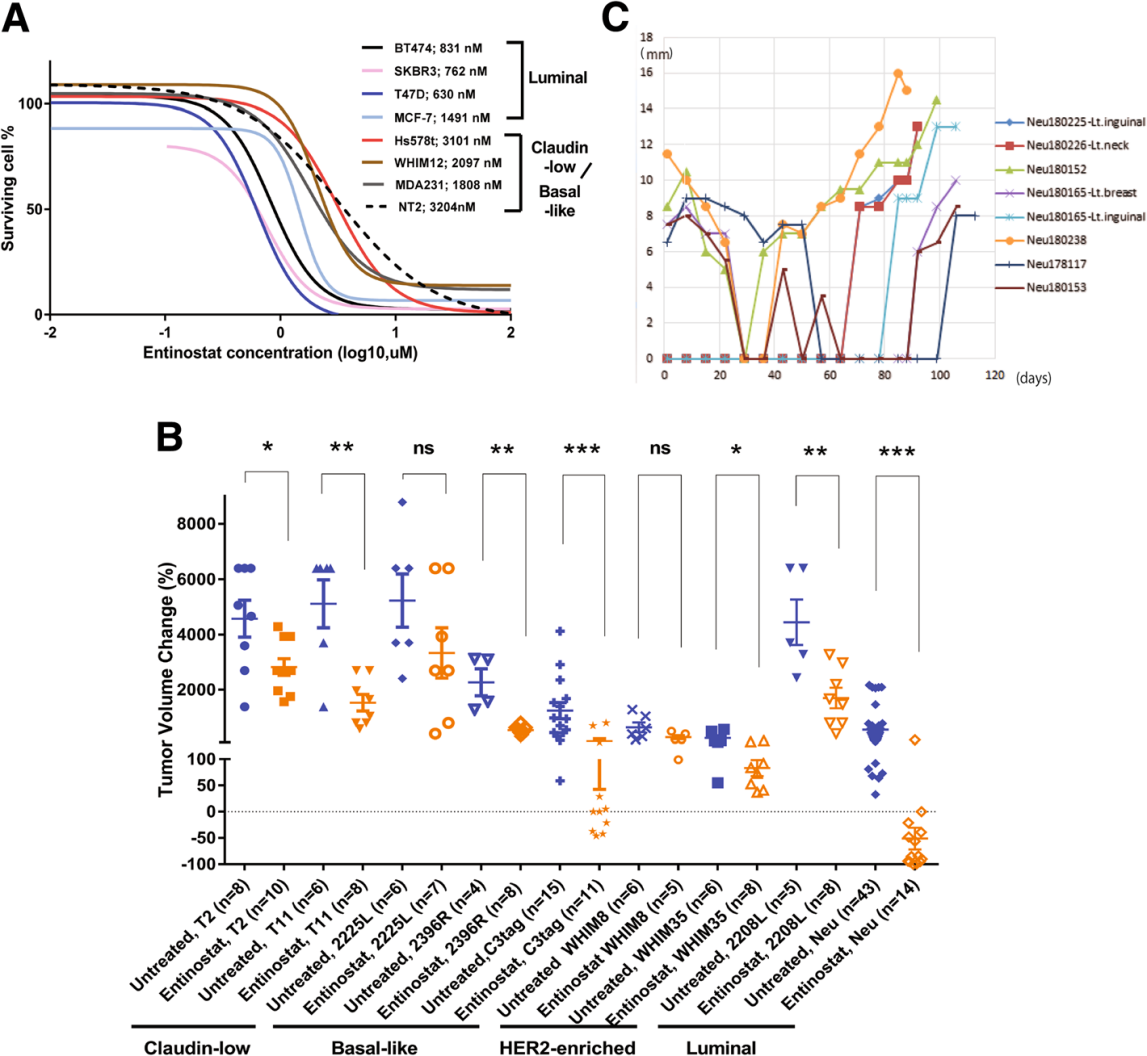
Luminal breast cancer is sensitive to entinostat in vivo and in vitro. **a** Antiproliferative activities of entinostat in breast cancer cells. The effects of entinostat on the proliferation of various human or mouse cancer cell lines were determined by using the [3-(4,5-dimethylthiazol-2-yl)-5-(3-carboxymethoxyphenyl)-2-(4-sulfophenyl)2H-tetrazolium [21] assay (CellTiter 96® AQueous One Solution Cell Proliferation Assay, Promega). Cells were seeded into 96-well culture plates and treated with entinostat for 3 days, and then treated with MTS for 2 h. Cell viability was determined by measuring the absorbance at 490 nm. Inhibitory concentration (IC) curves are shown with IC_50_ values in legend. **b** Short-term treatment responses for seven mouse models of mammary cancer. The models used were p53 null T2 and T11 which were chosen based on their similarity in gene expression to claudin-low subtype, 2396R, 2225L, and C3tag whose subtype was basal-like, WHIM8 and WHIM35 whose subtypes were HER2-enriched [24], and 2208L and MMTV/Neu whose subtypes were luminal [25]. Entinostat at 12 mg/kg was continuously administered via the chow. Tumor size was measured at baseline for all the models and at 7-day point for 2225L, 10-day point for 2396R and T2, 14-day point for 2208L, and 21-day point for T11, WHIM8, WHIM35, C3tag, and MMTV/Neu. Treatment periods were dependent on their faster growth rate. The change in tumor volume is plotted as whisker plots shown as measures of tumor responsiveness for entinostat-treated models with their matched untreated control. The error bars represent the 95% CIs. The number of animals in each treatment group is indicated in parentheses. Mann-Whitney tests were conducted between untreated control and entinostat-treated group for each mouse model. *, < 0.05; **, < 0.01; ***, < 0.001; ns, not significant. **c** A total of eight MMTV/Neu tumors from six mice showed complete response but finally became resistant to entinostat chow (12 mg/kg). 180226-Lt.Neck or 180165-Lt.inguinal were not detected at the beginning but appeared after 63 or 77 days of treatment, respectively

Given the promising phase II results for entinostat, our study sought to clarify the mechanism(s) of entinostat sensitivity and to find biomarker(s) to predict its sensitivity or resistance in human breast cancer. As a first step, we investigated preclinical models of breast cancer for entinostat sensitivity. To examine sensitivity in vitro, we identified the IC_50_ doses of entinostat for seven human breast cancer cell lines (Fig. 1a). Cell lines with luminal features (BT474, MCF-7, SKBR3, and T47D) were more sensitive to entinostat compared with basal-like or claudin-low (MDA231, Hs578t, and WHIM12) cell lines. Luminal model sensitivity was confirmed in vivo by testing murine luminal, basal-like, claudin-low TP53−/− GEMMs, and PDX models, with entinostat at 12 mg/kg (Fig. 1b). Entinostat generally inhibited tumor growth for most murine models, irrespective of subtype, but only the luminal MMTV/Neu murine tumors presented a complete reduction in tumor volume after treatment of entinostat. In addition, we assessed the effects of entinostat on the overall survival of tumor-bearing mice. Although entinostat generally prolonged the survival for mice with any of the tumor types, entinostat greatly extended lifespan from a median of 29 to 123 days in MMTV/Neu (Additional file 1: Figure S1). Figure 1d shows a time course of eight MMTV/Neu tumors from six mice; although they initially responded to entinostat completely, recurrent tumors emerged after the 5–10 weeks of entinostat treatment. In addition, we note some tumors (180226-Lt.Neck and 180,165-Lt.inguinal tumors) appeared after a long latency, perhaps suggesting initial repression of tumorigenic cells that emerged with acquired resistance.

### Global gene expression change caused by entinostat in luminal breast cancer

**Fig. 2:**
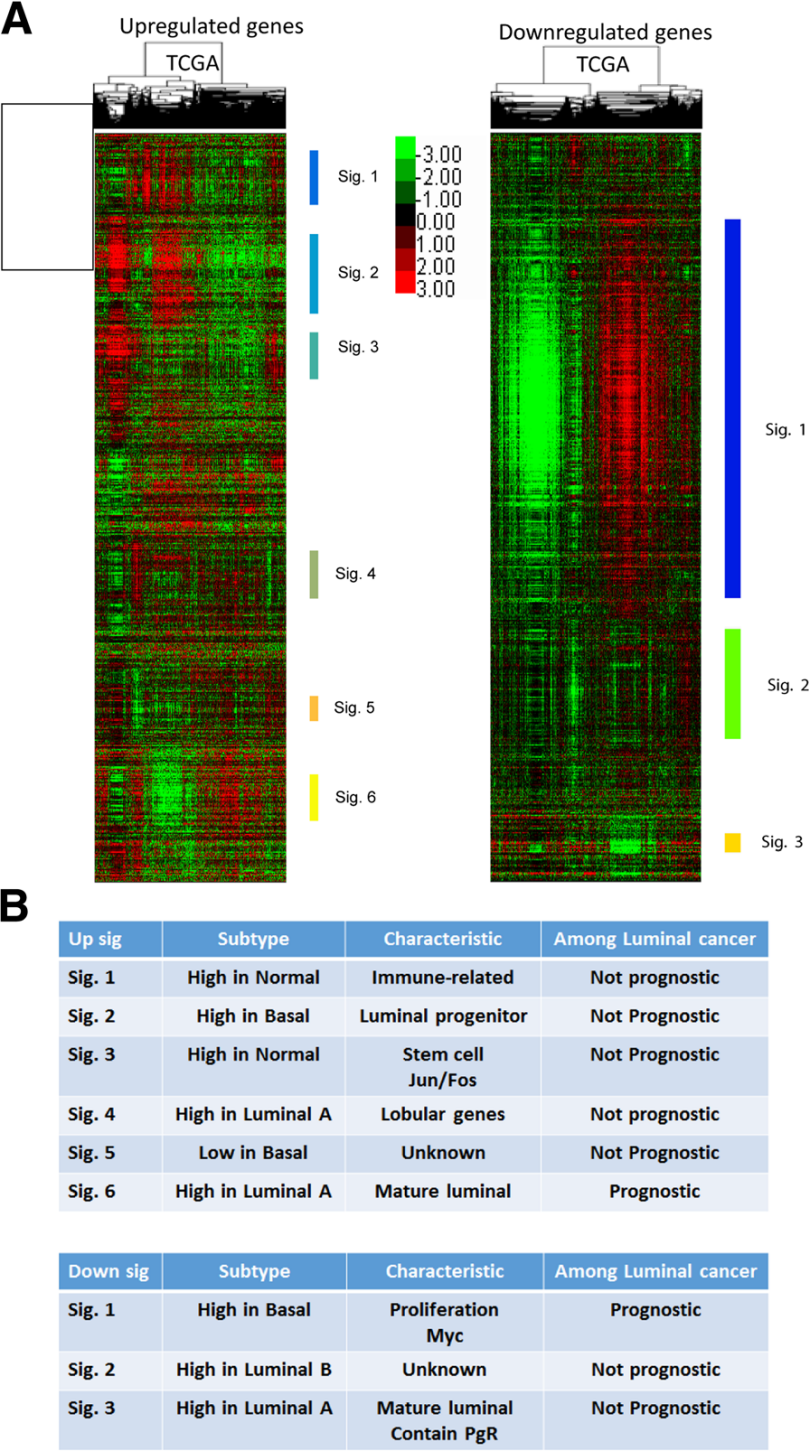
Entinostat inhibits luminal breast cancer through Myc signaling. **a** 563 upregulated and 565 downregulated genes with false positive rate (FDR) of 0% were hierarchically clustered using 817 TCGA breast cancer samples. We defined “signatures” as any gene set that contained a minimum of 10 genes and a Pearson node correlation greater than 0.5 in TCGA dataset. Using these criteria, we defined six and three signatures in upregulated and downregulated genes, respectively. **b** The summary of the characteristics of all signatures

To identify signatures of entinostat-regulated genes, gene expression analyses were performed. Luminal cell lines SKBR3 (ER-/HER2+), BT474 (ER+/HER2+), and MCF7 (ER+/HER2-) were treated with or without entinostat at their IC_50_ doses for 72 h and their gene expression profiles determined. Differential expression analysis between untreated vs entinostat-treated cell lines revealed 563 upregulated and 565 downregulated genes with FDR of 0% (Additional file 2: Table S2-A). We hierarchically clustered these two gene lists using 817 TCGA breast cancer samples, then defined “signatures” as any gene set that contained a minimum of 10 genes and a Pearson node correlation of greater than 0.5. Using these criteria, we defined six upregulated signatures and three downregulated signatures (Fig. 2a, all genes in signatures listed on Additional file 2: Table S2-B). We next investigated the potential impact of each signature on the survival among luminal breast cancer samples in METABRIC (Additional file 1: Figure S2-A). In addition, we plotted the scores for each entinostat signature according to PAM50 intrinsic subtypes using TCGA breast tumors (Additional file 1: Figure S2-B). Figure 2b shows the summary of the characteristics of all signatures. Within the downregulated gene signatures, down signature 1 (285 genes) had significant correlations with HALLMARK_MYC_TARGETS_v1 (q-value, 2.7e^− 17^) and HALLMARK_MYC_TARGETS_v2 (q-value, 1.7e^− 4^) in the GSEA “Investigate gene set” analysis. Similarly, when TCGA samples were scored for these entinostat signatures with 517 other signatures, clustering analysis revealed the down signature 1 was correlated with three Myc signatures (MYC.1PFDR_UP, MYC.2012, and DUKE.MODULE13) as well as many proliferation signatures (Pearson correlation, 0.64).

### Entinostat inhibits luminal breast cancer through Myc signaling

**Fig. 3:**
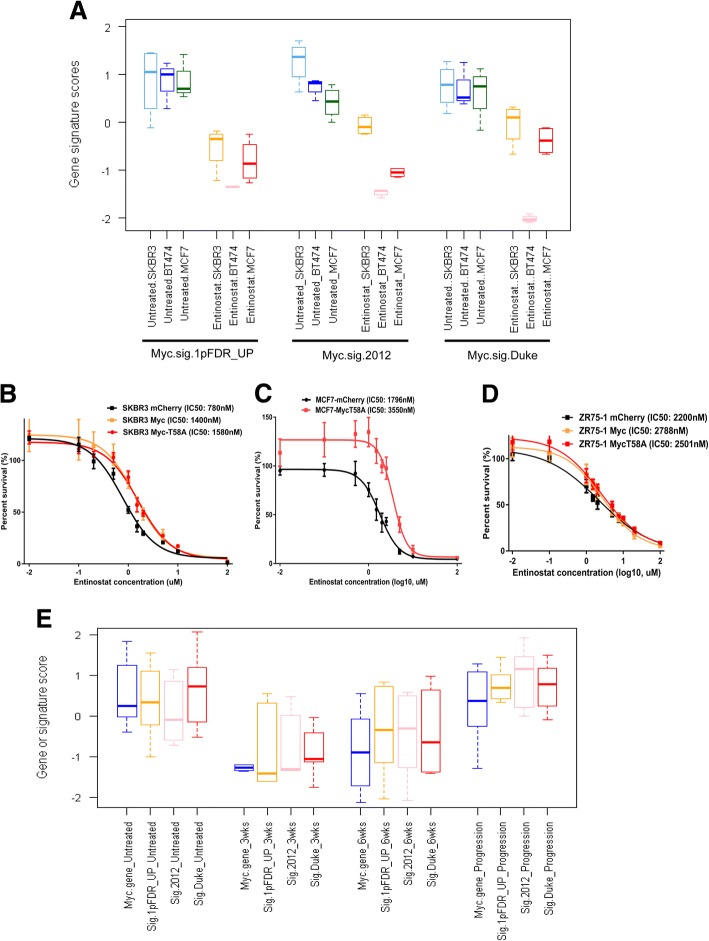
Myc signaling controls entinostat sensitivity. **a** Myc signature analysis of entinostat-treated human breast cancer cell lines. Box and whisker plots for the Myc gene signatures (Myc.sig.1PFDR_UP, Myc.sig.2012, or Myc.sig.DUKE) using the data from SKBR3, BT474, and MCF-7 treated with or without entinostat at their IC_50_ doses for 72 h. Each colored square represents the relative median transcript abundance (in log2 space) of each signature for untreated SKBR3, BT474, MCF-7 being light blue, blue or light green, or entinostat-treated SKBR3, BT474, MCF-7 being green, pink, or red. **b**–**d** Sensitivity to entinostat in luminal breast cancer cell lines with lentiviral *Myc* constitutive overexpression. After SKBR-3 (**b**), MCF-7 (**c**), or ZR75–1 (**d**) cells were transfected with lentivirus-mediated Myc shRNA, the cells were incubated in various doses of entinostat for 72 h, and then the viability of cells was measured using the MTS assay. Inhibitory concentration (IC) curves are shown with IC_50_ values in legend. Each point represents the mean ± standard deviation of sextuple determinations. Myc shRNA treatment makes luminal breast cancer cells more resistant toward entinostat as evidenced by an increase in IC_50_. **e** *Myc* gene expression (Myc_gene) or Myc signatures (Sig.1PFDR_UP, Sig.2012, Sig.Duke) in 27 MMTV/Neu luminal mouse model tumors untreated (*N* = 8) or treated with entinostat at 12 mg/kg for 3 weeks (*N* = 6), 6 weeks (*N* = 5), or until progression (*N* = 8)

Figure 3a illustrates that Myc signature scores goes down after treatment with entinostat at their IC_50_ doses in three luminal human cell lines. Myc is well appreciated as an oncogene that has been shown to induce HDAC2 (a major target of entinostat) in colorectal [47] and pancreatic cancer [12]; therefore, we sought to experimentally validate the role of Myc signaling in sensitivity to entinostat. Using lentiviral transfection of Myc or a phosphorylation incompetent mutant Myc^T58A^, we tested the impact of Myc overexpression on entinostat sensitivity. Western blot analysis confirmed Myc overexpression, and quantification revealed that protein levels of Myc were 3.2- and 16.5-fold higher than control cells in SKBR3 and ZR75-1, respectively. Similarly, Myc^T58A^ overexpressed cells had 5.4-, 1.4-, and 5.4-fold higher Myc protein levels than control cells in SKBR3, MCF7, and ZR75-1, respectively (Additional file 1: Figure S3-A & S3-C). Importantly, we observed that Myc and Myc^T58A^ overexpression in SKBR3, MCF-7, and ZR75-1 cells increased resistance to entinostat (Fig. 3b–d). Examining gene expression profiles of MMTV/Neu tumors responding to entinostat at a 3-week time point confirmed a reduction in expression of *Myc* and Myc target genes. Myc signature scores stayed in a low level at the 6-week point but increased when the tumors became resistant to entinostat (Fig. 3e, the changes in tumor size are shown in Additional file 2: Table S3). Further, *Myc* gene signatures did not change between 16 untreated vs 5 treated C3tag tumors, which did not respond to entinostat for 3 weeks (*p* = 0.99, data not shown). Taken together, these results demonstrate Myc as a possible target of entinostat therapy and point to repression of Myc target genes as a critical mediator of sensitivity.

### Genomic *Jun* loss causes resistance to entinostat

**Fig. 4:**
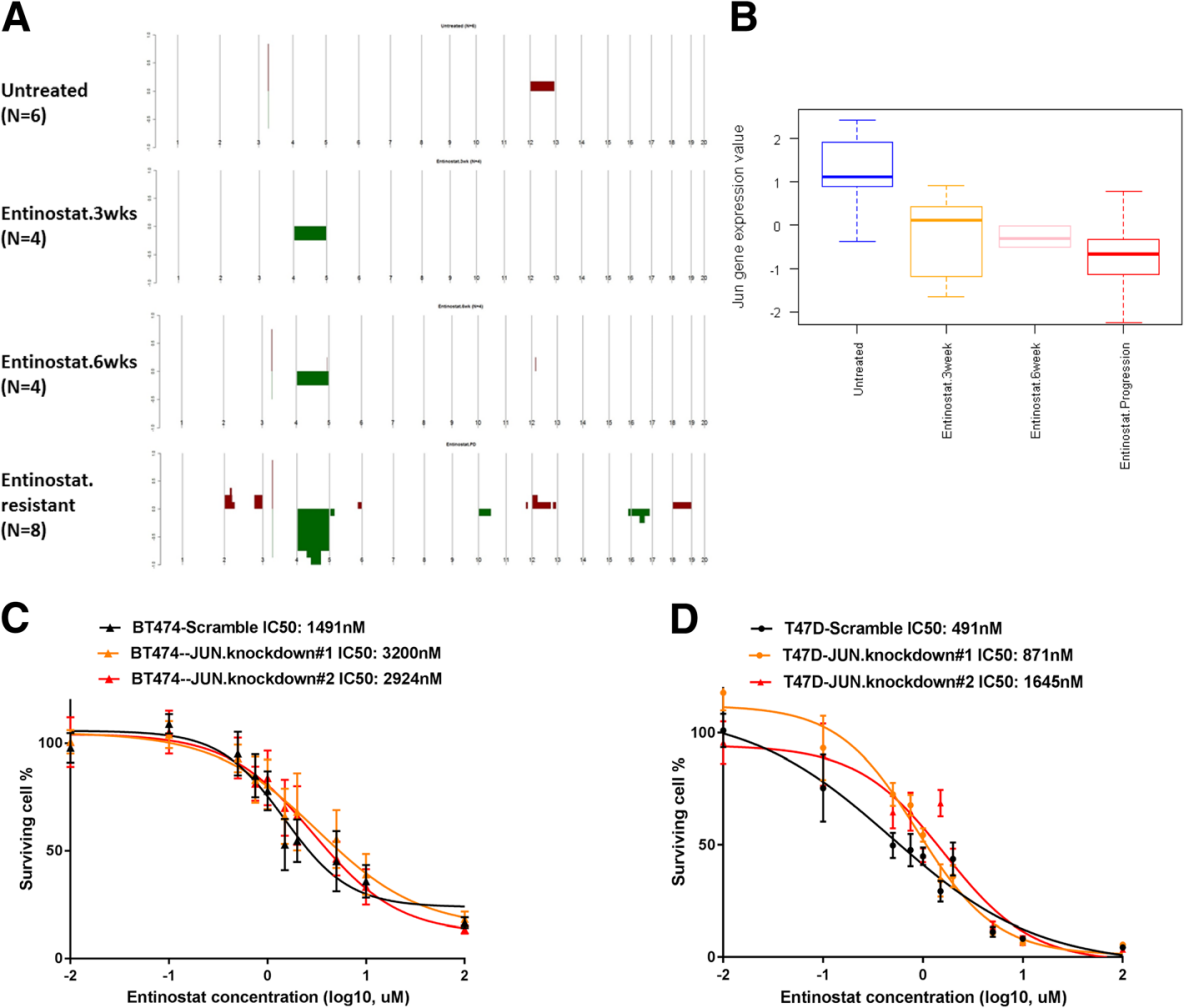
*Jun* copy number loss causes resistance to entinostat. **a** Copy number landscape of entinostat-treated MMTV/Neu tumors by arrayCGH. aCGH analysis revealed that a large portion of mouse Chromosome 4 had DNA copy number loss in tumors that progressed while on entinostat. Segments of copy number gains are plotted *above* the *x*-axis in *red* and loss are plotted *below* the *x*-axis in *green*. The frequency of alterations is indicated on the *y*-axis from 0 to 100%. **b** *Jun* gene expression in 27 MMTV/Neu luminal mouse model tumors untreated (*N* = 6) or treated with entinostat at 12 mg/kg for 3 weeks (*N* = 6), 6 weeks (*N* = 5), or until progression (*N* = 8). Each square represents the relative median transcript abundance (in log2 space) of each signature for untreated or entinostat-treated MMTV/Neu. **c**, **d** Sensitivity to entinostat in BT474 or T47D with lentiviral c-*Jun* knockdown. *Jun* shRNA treatment makes BT474 or T47D cells resistant toward entinostat as evidenced by an increase in IC_50_. After BT474 or T47D cells were transfected with lentivirus-mediated Jun shRNA, the cells were incubated in various doses of entinostat for 72 h and then the viability of cells was measured using the MTS assay. Each point represents the mean ± standard deviation of sextuple determinations. Inhibitory concentration (IC) curves are shown with IC_50_ values in legend

To identify potential mechanisms of resistance, we analyzed MMTV/Neu sensitive and resistant tumors by aCGH (Fig. 4a). Copy number aberration in untreated tumors was few. However, a large portion of mouse chromosome 4 showed DNA copy number loss in tumors that progressed while on entinostat for a long time. This observation led us to hypothesize that copy number loss at Chr.4 conferred resistance to entinostat. To test for driver genes responsible for the resistance, we used DawnRank analysis [40] to integrate copy number data with the gene expression data from MMTV/Neu tumors that were untreated, treated for 3 weeks, treated for 6 weeks, or treated until progression. Table 1 shows the DawnRank results where the top-ranked genes with copy number aberration are listed among MMTV/Neu tumors treated with entinostat. *Jun* was computationally identified to be a top driver gene with copy number loss associated with resistance/progression. Not only the ranking but also the percent rank of *Jun* gene increased from 0.977 at 3-week point (which means top 2.3% in the network) to 0.999 (top 0.1%) at the time of progression. We detected 83 genes, including *Jun*, at Chr.4, which significantly decreased their gene expression and DNA copy number levels with q-value 0% by supervised analysis comparing the untreated samples with the samples that became resistant while on entinostat (Additional file 2: Table S4). Of note, the entirety of samples resistant to entinostat showed significantly lower copy number in a region at Chr.4 between 80,385,673 and 101,147,931, where *Jun* is located. In addition, we note a reduction of *Jun* gene expression levels with the duration of entinostat therapy (Fig. 4b). To test the hypothesis that *Jun* copy number loss causes resistance to entinostat, we sought to determine whether *Jun* levels influence the effects of entinostat on cell viability. Lentiviral transfection of *Jun* shRNA reduced Jun protein expression levels by 54–70% in BT474 and T47D (Additional file 1: Figure S3-B & S3-C). In support of our hypothesis, reduction of Jun by shRNA imparted BT474 and T47D cells with increased resistance to entinostat (Fig. 4c, d).

**Table 1:** DawnRank analysis of entinostat-treated MMTV/Neu samples

Untreated (*N* = 6)			Entinostat-3wks (*N* = 4)			Entinostat-6wks (*N* = 4)			Entinostat-resistant (*N* = 8)		
Gene	Percent rank	Chr	Gene	Percent rank	Chr	Gene	Percent rank	Chr	Gene	Percent rank	Chr
MAX	1	12	JUN	0.977	4	JUN	0.997	4	JUN	0.999	4
CALM1	0.995	12	CDC42	0.975	4	CDC42	0.994	4	JAK1	0.995	4
HIF1A	0.990	12	JAK1	0.969	4	JAK1	0.992	4	CDC42	0.990	4
ACTN1	0.986	12	LCK	0.969	4	LCK	0.989	4	CDKN2A	0.989	4
FOS	0.981	12	LYN	0.965	4	LYN	0.987	4	LCK	0.987	4
HSP90AA1	0.976	12	MAP3K7	0.960	4	MAP3K7	0.984	4	TGFBR1	0.984	4
FOXA1	0.972	12	ERBB2	0.959	11	CDKN2A	0.982	4	LRP8	0.983	4
ARF6	0.967	12	CDKN2A	0.952	4	ZBTB17	0.979	4	ZBTB17	0.981	4
FBLN5	0.963	12	TGFBR1	0.950	4	TGFBR1	0.977	4	TLE1	0.980	4
NCOA1	0.958	12	RBBP4	0.948	4	RBBP4	0.974	4	LYN	0.980	4
NFKBIA	0.953	12	ZBTB17	0.944	4	LRP8	0.972	4	RBBP4	0.979	4

Chr, mouse chromosome; Entinostat-3wks, Entinostat-6wks, Entinostat-resistant are samples treated with entinostat for 3 weeks, 6 weeks, and until progression, respectively

### Genomic *Jun* copy number loss correlates Myc signaling activity

**Fig. 5:**
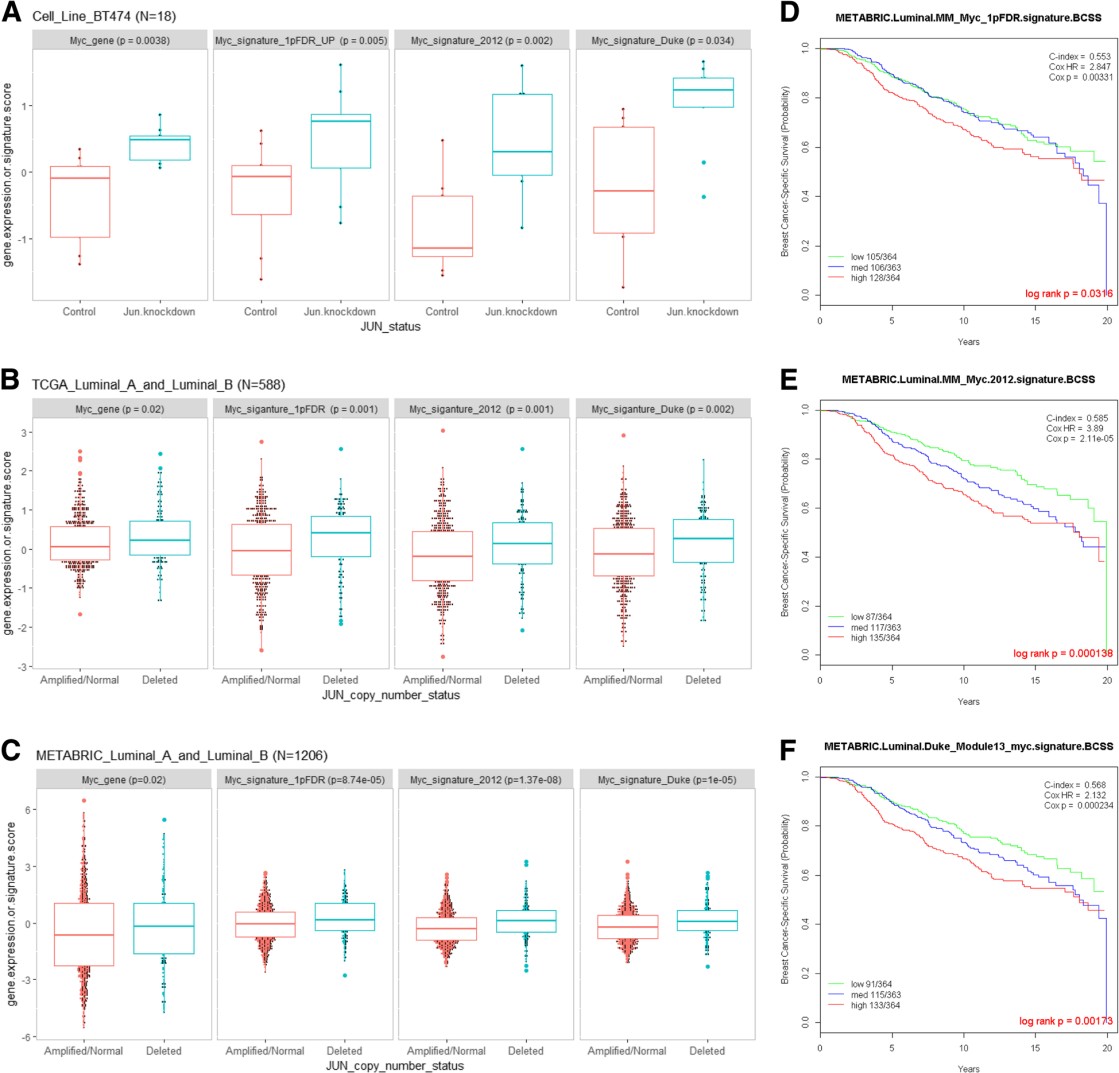
Genomic *Jun* copy number loss correlates Myc signaling activity. **a** Box and whisker plots are shown as *Myc* gene expression (*Myc*.gene) or signature (1PFDR_UP, 2012, DUKE) scores in BT474 with lentiviral c-*Jun* or scramble shRNA (Control). The middle bar in each square represents standardized, average values. The square represents the interquartile range (25th and 75th percentiles). **b** *Myc* gene expression or signature scores in luminal TCGA breast cancer samples with *Jun* deletion (*N* = 136) or without *Jun* deletion (amplified/normal, *N* = 452). **c** *Myc* gene expression or signature scores in luminal METABRIC breast cancer samples *Jun* deletion (*N* = 204) or without *Jun* deletion (amplified/normal, *N* = 1002). **d**–**f** Kaplan-Meier plots on breast cancer-specific survival in 1091 luminal breast cancer patients in METABRIC. Patients with survival of > 20 years were excluded. Patients were classified into three groups with lower, middle, or top third of the scores derived from the Myc signature scores using Myc_1pFDR (**d**), Myc.2012 (**e**), or Duke_Module13_myc (**f**). Two-sided log-rank tests and univariate Cox regression analyses were conducted to determine significance of each Myc signature

Given that sensitivity to entinostat was dependent on repression of Myc target genes and that resistance to entinostat was marked by *Jun* loss, we examined whether loss of c-Jun restored the molecular features of Myc activation. Suggesting coordination between Myc and c-Jun, we noted that *Myc* mRNA or signature levels increased as *Jun* copy number loss became deeper in MMTV/Neu samples while treated with entinostat (Fig. 3e and Fig. 4a). Therefore, we investigated the relationship between *Jun* copy number loss and Myc signaling in the luminal cell line BT474. *Jun* knockdown in BT474 correlated with upregulation of Myc gene or signature scores (Fig. 5a). To test whether our observations in preclinical models might extend to human breast cancer patients, we examined *Jun* copy number in human breast cancer datasets. *Jun*-deleted human tumors had significantly higher levels of Myc gene or signatures among both TCGA and METABRIC luminal breast cancer samples (Fig. 5b, c). All three Myc signatures were prognostic among luminal breast cancer samples in METABRIC as well (Fig. 5d–f), and multivariable analyses accounting for age, tumor size, nodal status, and HER2 status showed two out of three Myc signatures were still significantly prognostic (Additional file 2: Table S5). Collectively, these results suggest that genomic DNA *Jun* copy number loss allows activation of Myc signaling, which in turn promotes resistance to entinostat therapy.

### Clinical impact of *Jun* copy number loss in human breast cancer datasets

**Fig. 6:**
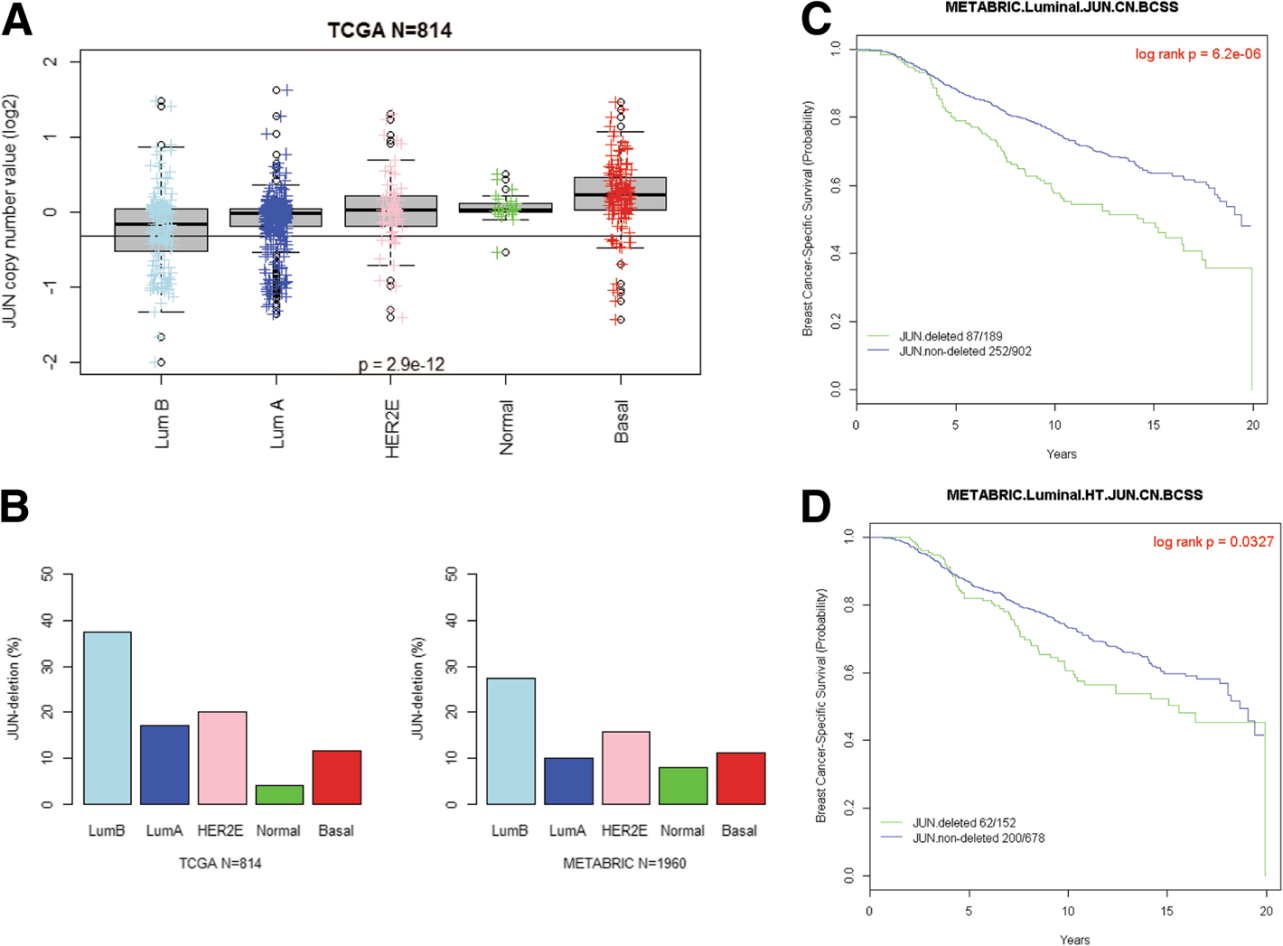
Clinical impact of *Jun* copy number loss in human breast cancer datasets. **a** Log2-based value of *Jun* copy number across the intrinsic subtypes in 814 TCGA breast cancer patients. Whiskers represent the 5%–95% distribution, boxes represent the interquartile range (25th and 75th percentiles), and the horizontal line in the box represents the median value. Cut-off value of *Jun* copy number for deletion was − 0.32. ANOVA *p* value was calculated by comparing *Jun* copy number values across all subtypes. **b** Frequencies of samples with *Jun* deletion in TCGA (*n* = 814) or METABRIC (*n* = 1960) according to the intrinsic subtype. **c**, **d** Kaplan-Meier plots on breast cancer-specific survival among luminal breast cancer patients in METABRIC. Patients with survival time of > 20 years were excluded. **c** Whole luminal A and B breast cancer patients (*N* = 1091). Cox *p* value = 8.12e−06. **d** Luminal breast cancer patients who received hormonal therapies (*N* = 830). Cox *p* value = 0.033

*Jun* DNA copy number values are generally lower in patients with luminal A and luminal B breast cancers (Fig. 6a). The frequencies of *Jun* copy number loss in luminal breast cancer were 23% and 17% in TCGA and METABRIC, respectively (Fig. 6b). Patients with *Jun* copy number loss had a worse prognosis among luminal breast cancer samples in METABRIC (Fig. 6c). Further, patients with *Jun* copy number loss who received hormonal therapies also had worse prognosis compared with similar patients without *Jun* copy number loss (Fig. 6d). We additionally performed survival analysis according to *Jun* copy number status and each Myc signature among METABRIC patients with luminal breast cancer who received hormonal therapies (Additional file 1: Figure S4). Patients with Jun-deleted/high Myc signature score had the worst prognosis (light green line), while patients with Jun-non-deleted/low Myc signature scores had the best prognosis (pink line). These results suggest that *Jun* copy number loss is frequently observed in human breast cancer patients, is prognostic of worse outcomes, and might be a genetic cause of resistance to entinostat among patients with luminal tumors who receive hormonal therapies.

## Discussion

With our work, we identify a complex interplay between the HDAC inhibitor entinostat, c*-Jun*, and *Myc*. Preclinical models, both human and murine, of luminal subtypes exhibited sensitivity to entinostat compared to basal-like or claudin-low subtypes. Importantly, we show that entinostat inhibited luminal breast cancer through Myc signaling, and genomic *Jun* loss upregulated Myc signaling to promote resistance to entinostat. Our findings indicate that *Jun* copy number loss might, therefore, represent a useful biomarker for entinostat resistance in luminal breast cancer where these *Jun*-deleted patients might be suggested to not receive entinostat containing regimens. Another alternative biomarker for entinostat resistance could also be high Myc signature expression. These novel findings are to the best of our knowledge, have not been previously reported.

*Myc* is the most frequently amplified oncogene [48] and it is known to regulate transcription of genes involved in cell growth and proliferation [49, 50]. The functions of Myc is influenced by multiple mechanisms in tumor cells: protein ubiquitination, gene amplification, chromosomal translocation, mutation, co-factor expression, and mutation of upstream signaling pathways [51–55]. Therefore, transcriptional or genomic levels of *Myc* itself do not always reflect on its activity. Gene signatures of Myc target genes might better reflect on Myc activity; however, Myc amplifies thousands of actively transcribed genes within each cell type, so every cancer has a different, specific cohort of *Myc* target genes [56, 57]. This is the reason why we investigated a number of different *Myc* signatures, all of whom were derived from breast epithelial tissues, and all were prognostic in patients with luminal breast cancer (Fig. 6). In our study, Myc signatures were repressed while breast epithelial cells were responsive on entinostat, and Myc signatures were reactivated upon progression in vivo. Myc signatures were repressed after entinostat treatment at IC_50_ doses and Myc overexpression resulted in resistance to entinostat in vitro. These data support the hypothesis that Myc represents an important modulator for response to entinostat as extensive evidence on other HDAC inhibitors has suggested [12–15].

This study also revealed that a specific region of mouse chromosome 4 was recurrently deleted in every entinostat-resistant MMTV/Neu tumor obtained (*n* = 8), but not in untreated tumors. This deletion has been reported in a variety of luminal breast cancer mouse models like MMTV/Neu and p53 null luminal tumors [25, 39, 58, 59]. Major human counterparts of mouse chromosome 4 are Chr.1p31-36 and Chr.9; loss of heterozygosity on 1p (where c-Jun is) occurred preferentially in a subclass of estrogen receptor-positive breast cancers [60] and has been shown as a poor prognostic factor [61–63]. These findings imply that deleted mouse chromosome 4 drives tumor aggressiveness, and this is a region also linked to poor outcomes in human luminal breast cancers as well.

To identify possible drivers in this luminal tumor conserved region of deletion, we used a network-based, integrated bioinformatics analysis (i.e., DawnRank) that identified *Jun* deletion at mouse chromosome 4 as the top driver gene upon progression during entinostat treatment. Knockdown of *Jun* expression in luminal cells increased resistance to entinostat (Fig. 4c, d), and genomic loss of *Jun* at 1p32, which was found in 17–23% of patients with luminal breast cancer, was significantly prognostic among patients with luminal breast cancer who received anti-hormonal therapies (Fig. 6). Furthermore, we found *Jun*-deleted samples had higher Myc signature scores in vitro and in vivo in human breast cancer (Fig. 5). Similar findings have been found between loss of heterozygosity of chromosome 1p32-pter and amplification of *Myc* [64]. The precise molecular mechanism(s) as to how *Jun* deletion causes upregulation of Myc signaling remains unclear, but the interplay between Jun and Myc has been previously reported [65, 66]. In detail, Jun/Ap-1 complex might regulate Myc directly [65] or Jun loss may modulate Myc function indirectly [66]. We would propose a mechanism that genomic *Jun* loss constitutively activates Myc signaling, which leads to poor outcomes in general, and possible resistance to entinostat. Measuring Jun DNA copy number loss is one of the candidate biomarkers for a clinical test, if entinostat achieves approval. Alternatively, gene expression of Myc signatures are also a clinical test candidate; those who have high Myc signature would be predicted to have resistance to entinostat. Lastly, we will need retrospective analysis of existing clinical trials to determine which one is the best.

## Conclusions

Entinostat inhibited luminal breast cancer through Myc signaling, which was upregulated by *Jun* DNA loss to promote resistance to entinostat in our models. Here we also provide a testing platform using MMTV/Neu with genomic Jun loss for combination therapies with entinostat to provide more durable response to *Jun*-deleted human luminal breast cancer. Further studies will be certainly required to validate the significance of genomic *Jun* loss in prospectively collected luminal breast cancer samples under treatment of entinostat.

## Additional files

Additional file 1: **Figure S1.** Long-term survival results for eight mouse models of basal breast cancer. **Figure S2.** Analysis of potential entinostat gene signatures. **Figure S3.** Western blotting results on lentiviral transfection of Myc or shJun. **Figure S4.** Clinical impact of *Jun* copy number loss in METABRIC. (DOCX 8120 kb)

Additional file 2: **Table S1.** Genes in mouse and human version of Myc signatures. **Table S2.** A. Upregulated and downregulated genes with FDR of 0% in SAM analysis. B: Entinostat signatures. **Table S3.** The changes in tumor size of MMTV/Neu tumors treated with entinostat. **Table S4.** 83 genes which significantly decreased at gene expression and copy number levels with q-value 0% by SAM analysis comparing the untreated samples and the samples resistant while on entinostat. Table S5. Multivariate analysis of each Myc signature accounting for clinical features in METABRIC. (XLSX 74 kb)
