# Supplementary material for: Identification of Jun loss promotes resistance to histone deacetylase inhibitor entinostat through Myc signaling in luminal breast cancer
Source: Genome Med. 2018 Nov 30;10:86. doi: 10.1186/s13073-018-0597-3 (PMC6267061; doi:10.1186/s13073-018-0597-3)
Supplement: Supplementary file 1 — Figure S1. Long-term survival results for eight mouse models of basal breast cancer. Figure S2. Analysis of potential entinostat gene signatures. Figure S3. Western blotting results on lentiviral transfection of Myc or shJun. Figure S4. Clinical impact of Jun copy number loss in METABRIC. (DOCX 8120 kb) [file 13073_2018_597_MOESM1_ESM.docx]

**
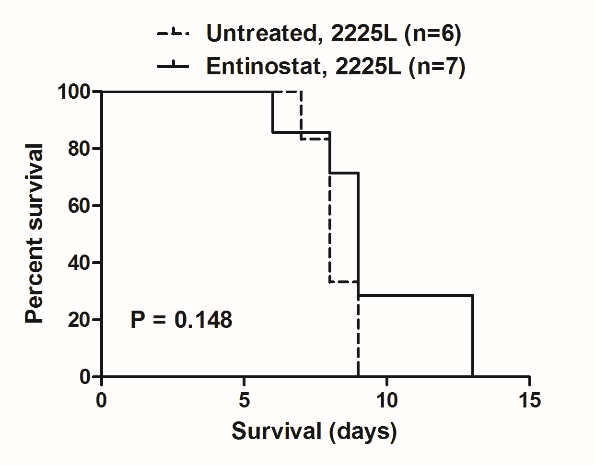

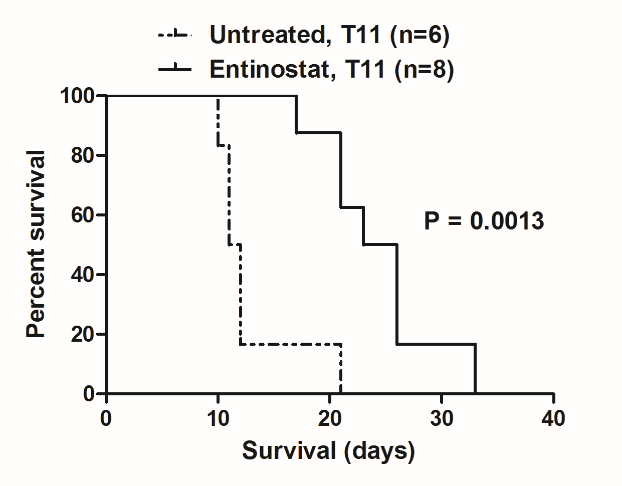

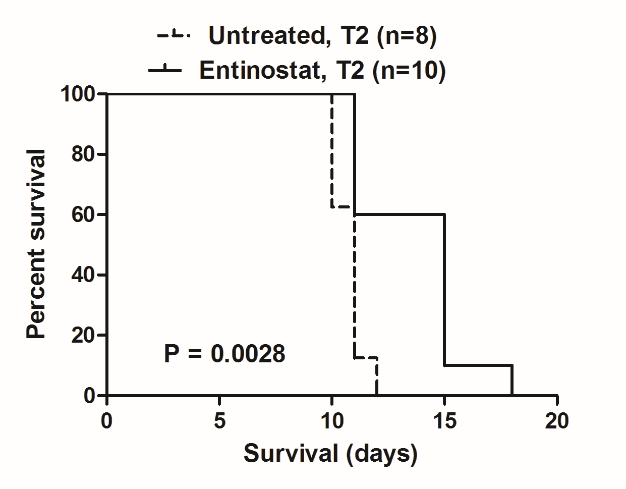
**

**
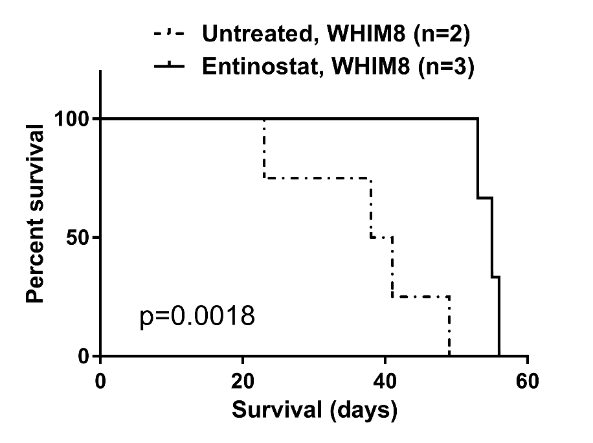

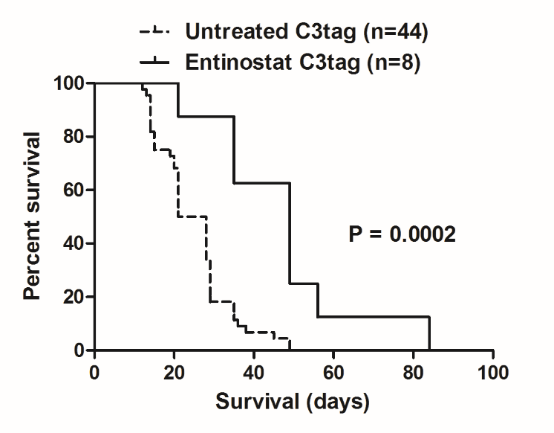

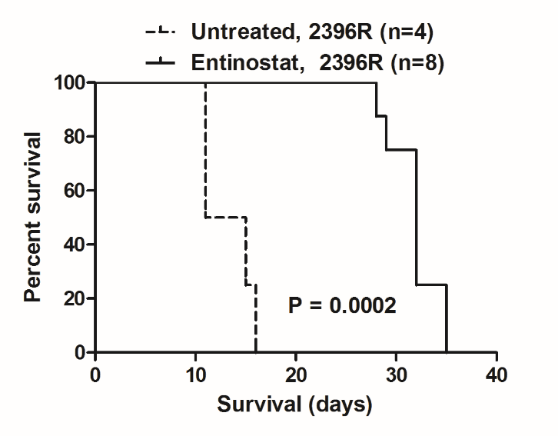
**

**
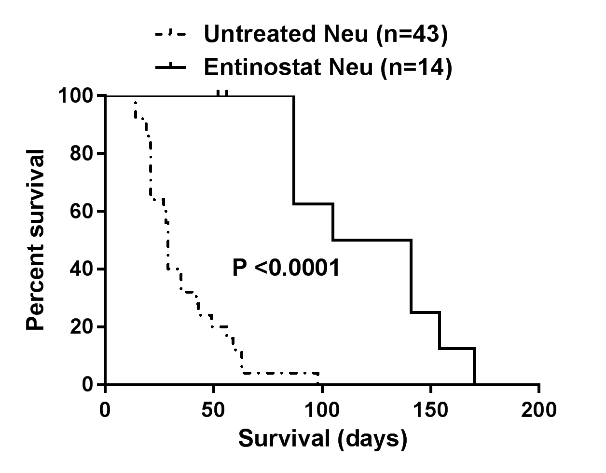
**

**
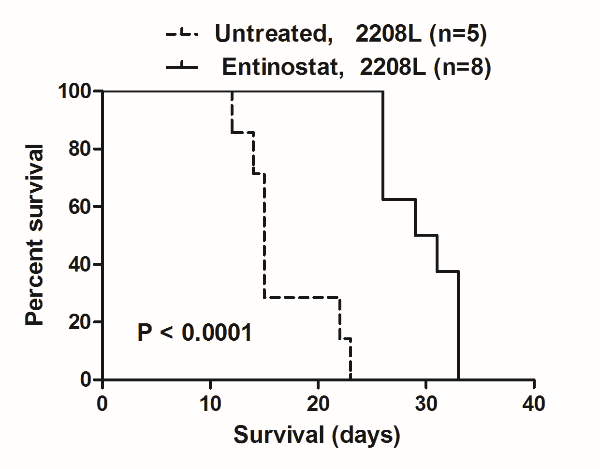

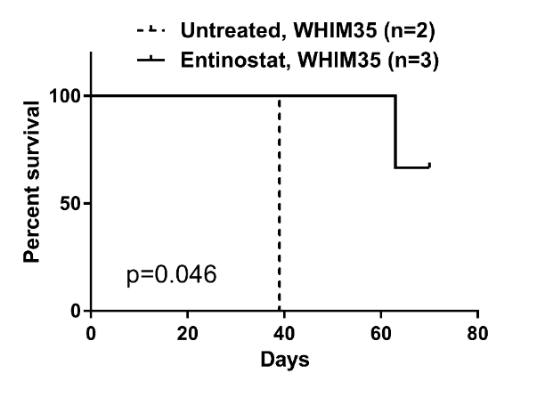
**

**Supplementary Figure S1. Long-term survival results for eight mouse models of basal breast cancer.** Kaplan–Meier analyses on overall survival of were conducted for untreated control and Entinostat-treated groups. A two-sided log-rank test was conducted to determine signiﬁcance of the treatment with Entinostat.


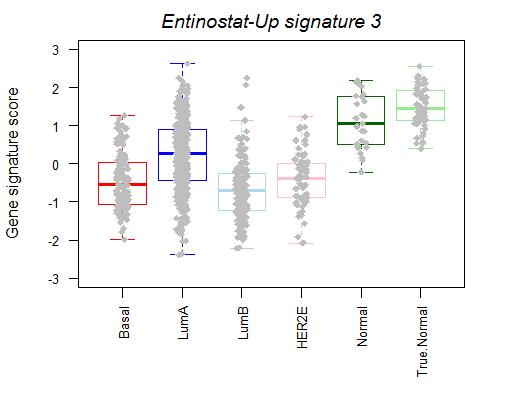

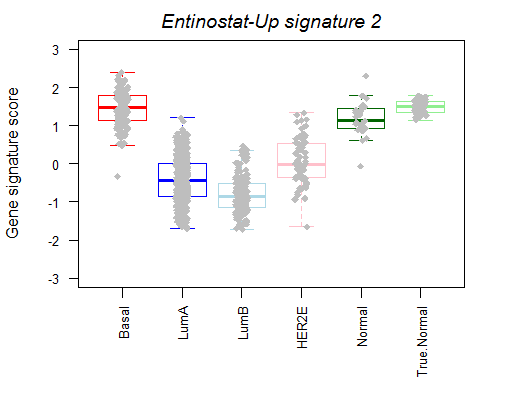

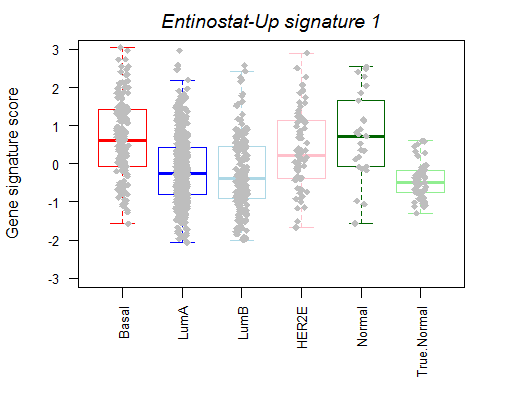
**A**


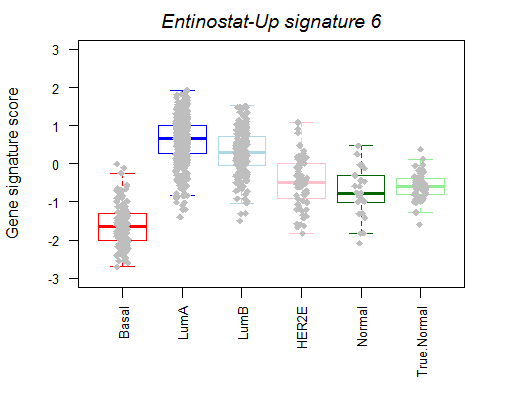

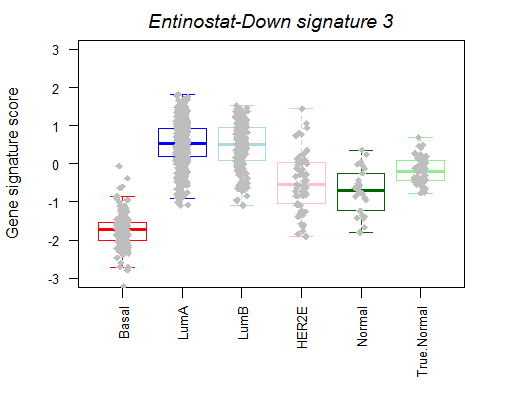

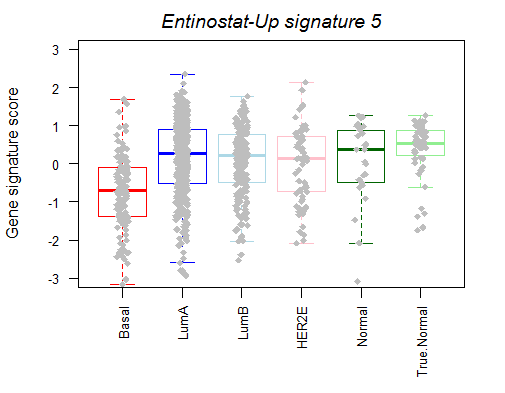

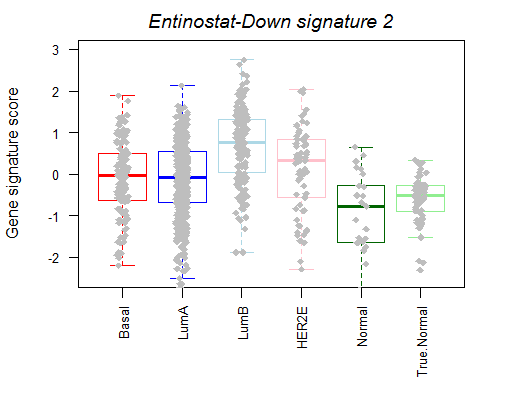

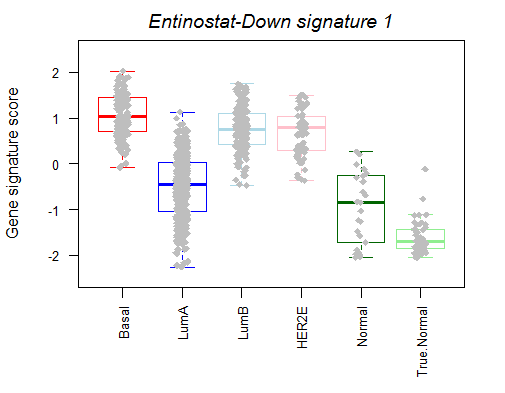

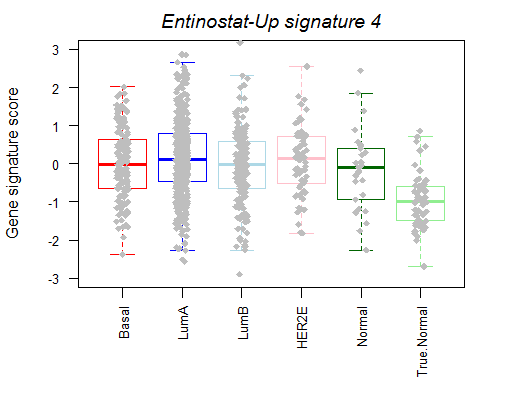


**
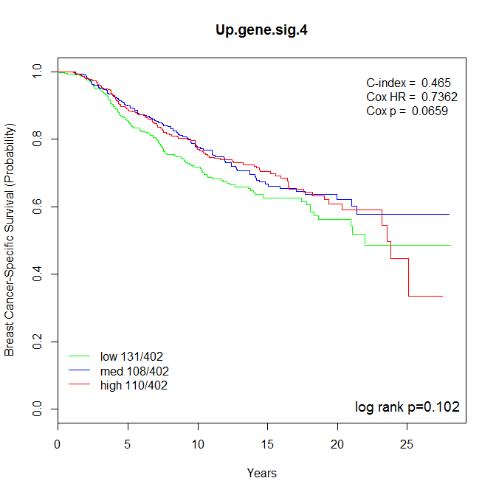

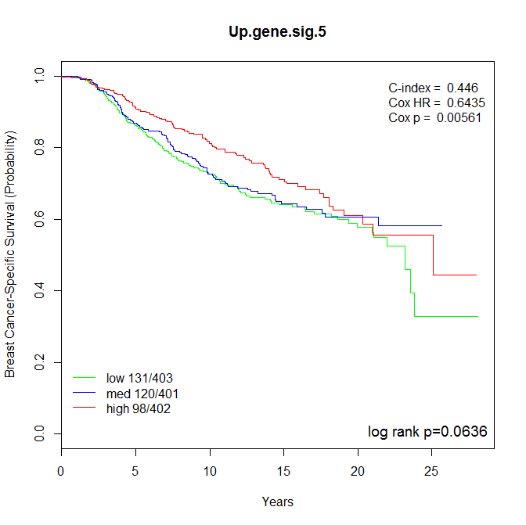

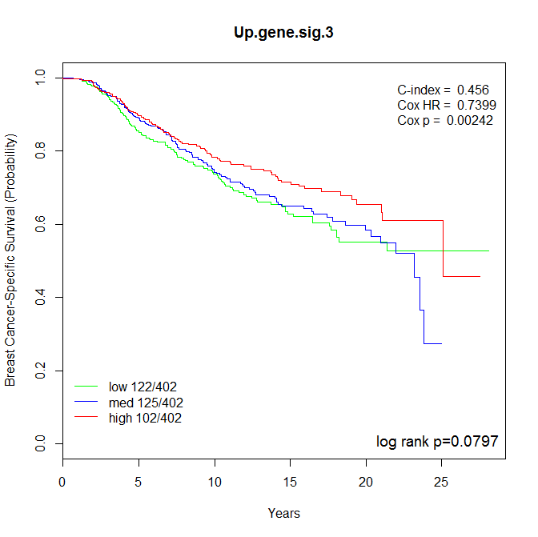

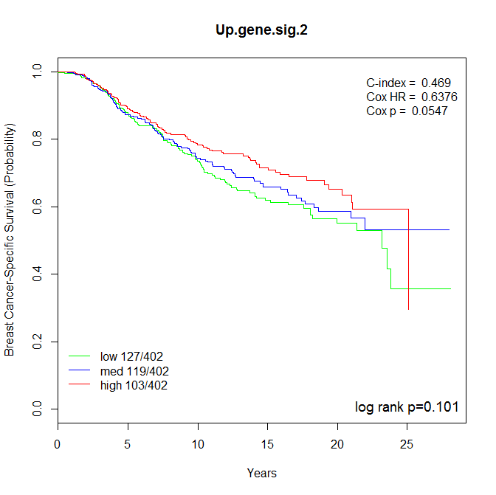

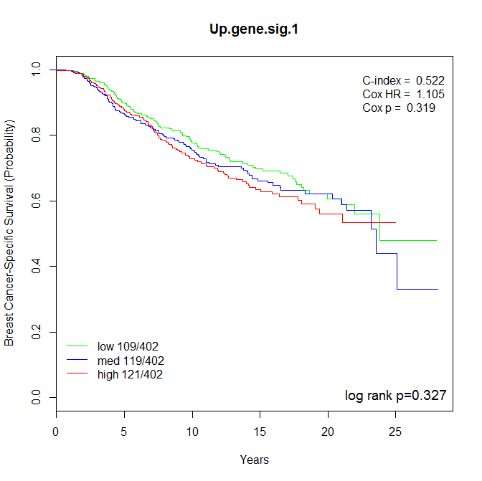

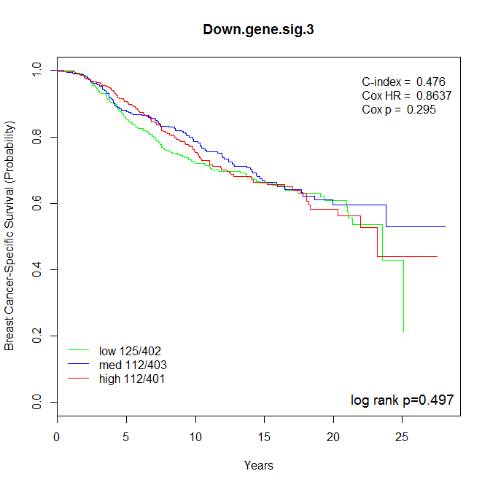

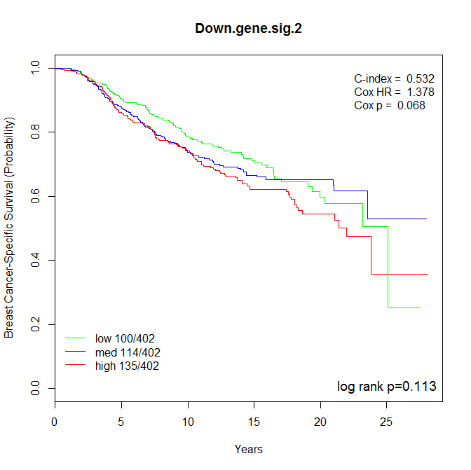

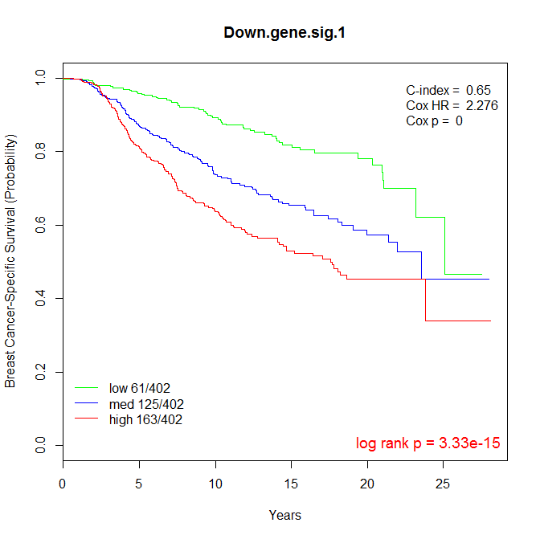

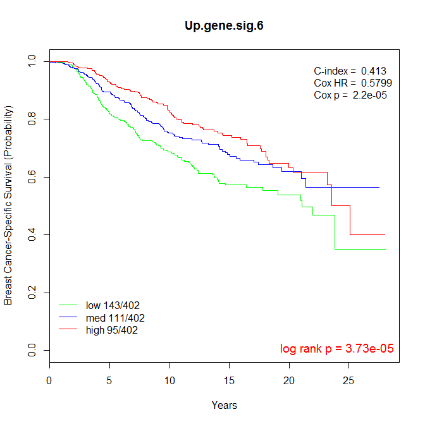
B**

**Supplementary Figure S2.** **Analysis of potential Entinostat gene signatures. (**A) A mean expression value of the genes in each signature for each patient in METABRIC was determined. The METABRIC patients were separated into three equal groups representing low, medium and high average expression for each signature. Survival analyses were performed using log-rank tests. (B) The scores for each Entinostat signature were plotted according to the intrinsic subtypes in 817 TCGA breast cancer samples. “True-Normal” means non-cancer normal tissue to differentiate from Normal(-like) subtype.

**
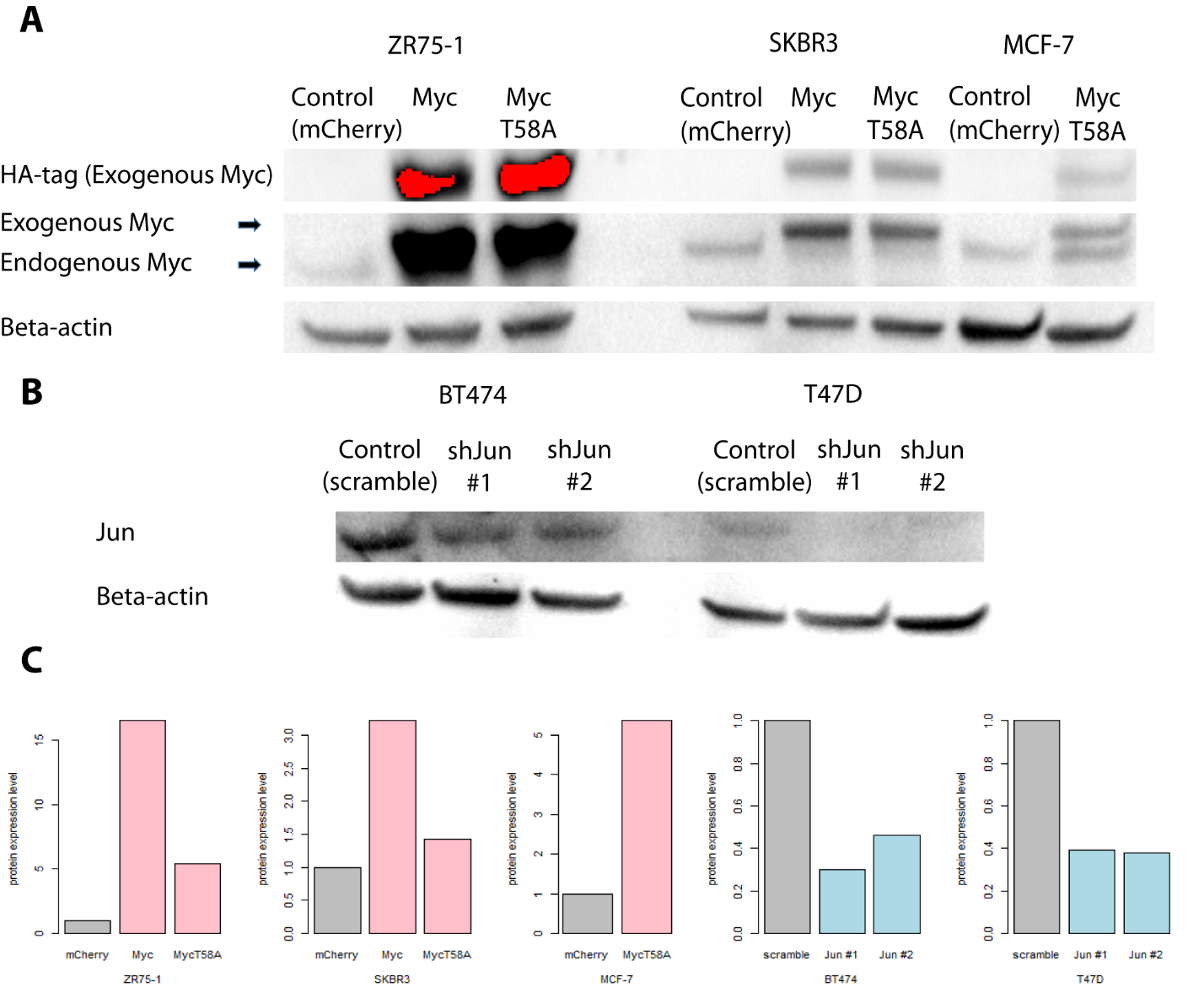
**

**Supplementary Figure S3. Western blotting results on lentiviral transfection of Myc or shJun.** (A) SKBR-3, ZR75-1, and MCF-7 were transfected with lentiviral mCherry (control), *Myc*, or mutant *Myc* (T58A) (B) BT474 and T47D were were transfected with lentiviral scramble shRNA (control) or 2 kinds of sh*Jun*  (C) The relative chemiluminescent intensities were quantified in individual frames between mCherry, Myc , and mutant Myc or between shRNA (control) and 2 kinds of sh*Jun*

**
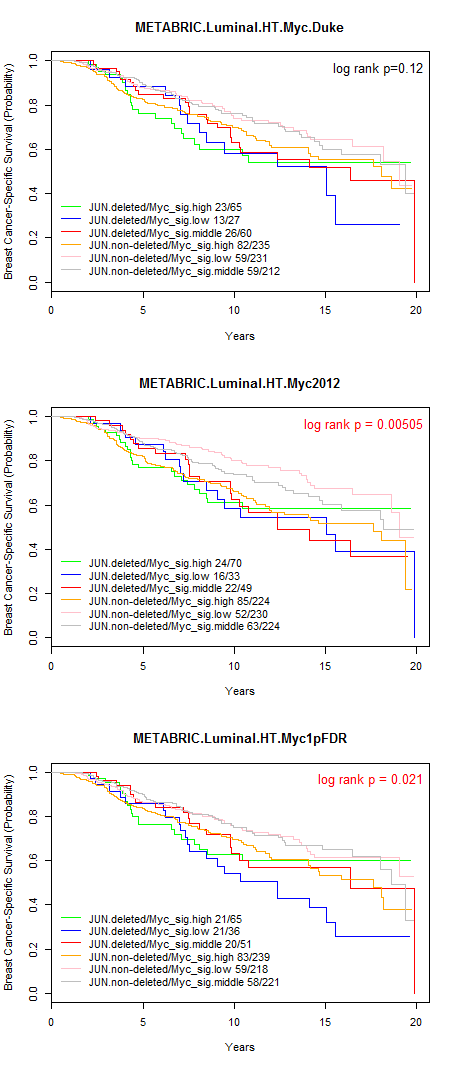
**

**Figure S4. Clinical impact of *Jun* copy-number loss in METABRIC.　S**urvival analysis according to *Jun* copy number status and each Myc signature among METABRIC patients with Luminal breast cancer who received hormonal therapies. Upper, Myc signature Duke; Middle, Myc signature 2012; Lower, Myc signature 1PFDR_UP. Patients with Jun-deleted / high Myc　signature score had the worst prognosis (light green line), while patients with Jun-non-deleted / low Myc signature scores had the best prognosis (pink line).
